# Bioinformatics Methods for Mass Spectrometry-Based Proteomics Data Analysis

**DOI:** 10.3390/ijms21082873

**Published:** 2020-04-20

**Authors:** Chen Chen, Jie Hou, John J. Tanner, Jianlin Cheng

**Affiliations:** 1 Department of Electrical Engineering and Computer Science, University of Missouri, Columbia, MO 65211, USA; ccm3x@mail.missouri.edu; 2 Department of Computer Science, Saint Louis University, St. Louis, MO 63103, USA; jie.hou@slu.edu; 3 Program in Bioinformatics & Computational Biology, Saint Louis University, St. Louis, MO 63103, USA; 4 Departments of Biochemistry and Chemistry, University of Missouri, Columbia, MO 65211, USA; tannerjj@missouri.edu

**Keywords:** bioinformatics analysis, computational proteomics, machine learning

## Abstract

Recent advances in mass spectrometry (MS)-based proteomics have enabled tremendous progress in the understanding of cellular mechanisms, disease progression, and the relationship between genotype and phenotype. Though many popular bioinformatics methods in proteomics are derived from other omics studies, novel analysis strategies are required to deal with the unique characteristics of proteomics data. In this review, we discuss the current developments in the bioinformatics methods used in proteomics and how they facilitate the mechanistic understanding of biological processes. We first introduce bioinformatics software and tools designed for mass spectrometry-based protein identification and quantification, and then we review the different statistical and machine learning methods that have been developed to perform comprehensive analysis in proteomics studies. We conclude with a discussion of how quantitative protein data can be used to reconstruct protein interactions and signaling networks.

## 1. Introduction

Proteins are essential molecules in nearly all biological processes. They provide the structural scaffolding in cells, and function in myriad processes, such as metabolism, biosignaling, gene regulation, protein synthesis, solute transport across membranes, immune function, and photosynthesis. Abnormal regulation of protein function is one of the most prominent factors in disease pathologies; thus, understanding how the proteome is perturbed by disease is an important goal of biomedical research. It is well known that transcriptome data, such as mRNA abundance, is insufficient to infer the protein abundance [1,2], and thus, direct measurements of protein activities are often necessary. Traditional approaches tend to focus on one or a few proteins; however, with the recent developments in the sample separation and mass spectrometry technologies, it is now possible to consider a complex biological system as an integrated unit. The rapid advancements in the experimental aspects of proteomics have inspired various downstream bioinformatics analysis methods that help to discover the relationship between molecular-level protein regulatory mechanisms and phenotypic behavior, such as disease development and progression [3,4].

**Figure 1:**
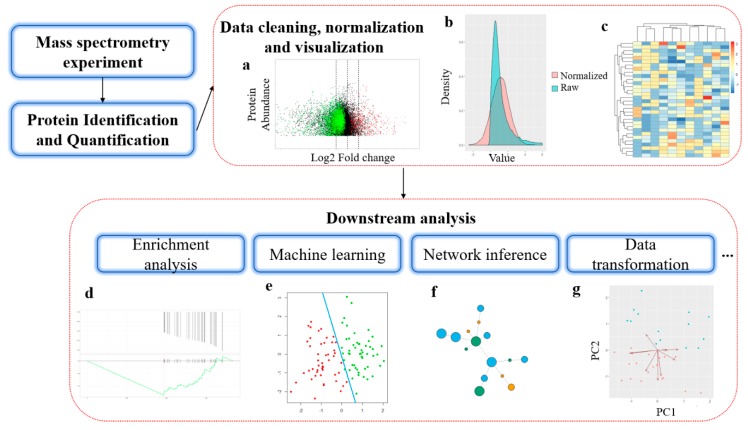
General workflow of bioinformatics analysis in mass spectrometry-based proteomics. (**a**) MA-plot from protein differential abundance analysis. X-axis is the log2 transformed fold change and Y-axis is the average protein abundance from replicates. (**b**) Distribution of protein abundance data before and after normalization. (**c**) Heatmap for protein abundance with clustering; (**d**) Protein set enrichment analysis, Y-axis in the above plot shows the ranked list metric, and in the bottom plot shows the running enrichment score. X-axis is the ranked position in protein list. (**e**) Machine learning-based sample clustering. (**f**) Illustration of a network inferred from proteomics data. (**g**) Dimensionality reduction of proteomics expression profile.

The typical experiment strategy for MS-based proteomics can be divided into two broad categories based on the size of the protein analyzed by MS: bottom-up and top-down [5]. In the more common bottom-up approach, the protein samples are first proteolytically digested into peptides before analyzing in a mass spectrometer [6]. In top-down proteomics, intact proteins are directly analyzed by MS [7,8]. In this review, we mainly focus on bioinformatics software and platforms designed for protein identification, quantification, and downstream analysis in bottom-up proteomics. The downstream analysis refers to various data analysis methods used to extract biological meaning from protein abundance data from MS experiments [9]. Similar to genomics, these bioinformatics methods are in rapid development and often require interdisciplinary efforts from mathematicians, statisticians, and computer scientists. Figure 1 shows the general workflow of bioinformatics analysis in mass spectrometry-based proteomics.

In this review, we first describe the tools and methods used to process the raw mass spectral data, including identification and quantification of peptides and proteins. In the following sections, we discuss various bioinformatics techniques used to process the proteomics data. Since the downstream analysis of proteomics does not have a standard workflow and can be highly specific to a particular research purpose, we first introduce the algorithms and tools used in three major applications: data preprocessing, statistical analysis, and enrichment analysis. Then we discuss popular machine learning methods and how they are applied to specific biomedical research topics in proteomics. Finally, we discuss how proteomics data can be used to reconstruct protein interaction and signaling networks. It is beyond the scope of this review to describe all the bioinformatics methods that have developed for proteomics analysis. Therefore, we will focus on the most popular software tools that are in use, as well as some novel methods that have been developed for emerging new experimental technologies.

## 2. Mass Spectrometry-Based Protein Identification and Quantification

### 2.1. Peptide and Protein Identification

After fragmentation spectra data from MS are acquired, the first procedure is to determine the sequence of the peptides. Two different strategies can be applied to this task: searching against the fragmentation spectra databases [10,11,12] and de novo peptide sequencing [13,14,15]. Table 1 lists a selection of software tools commonly used in peptide and protein identification reviewed in this study.

In the database matching approach, a target database is established from in silico digestion of all expressed or hypothetical protein sequences. Then a peptide spectrum match (PSM) score is calculated for each fragmentation spectra and all theoretical fragmentation spectra information from the target database. The peptide that has the highest PSM score can be used as a candidate for the query peptide. It is always a crucial task to choose appropriate searching algorithms that can yield high quality peptide spectrum matching results from databases. Traditional protein database search engines, such as SEQUEST [16], implement a scoring function based on normalized cross-correlation of the mass-to-charge ratio predicted from known amino acid sequences in databases and the fragment ions detected from the tandem mass spectrum. MASCOT [17] is another popular software developed later, which integrates mass measurements with protein sequence information, peptide molecular weights from protein digestion and tandem mass spectrometry data to generate a probability-based score for protein identification.

Choosing appropriate input parameters is key for database searching. The tolerances for precursor mass and fragment mass are two important parameters, which should be chosen wisely. The former controls the peptide candidates considered for each spectrum, while the fragment mass tolerance controls the upper limit of the absolute value of the difference between the detected and theoretical fragment masses for a match. For both parameters, setting values that are too narrow will exclude possible true PSMs, while allowing the values to be too wide will introduce a large amount of false PSMs. Several methods have been developed to optimize these database search parameters. They often rely on the observed m/z values of known ions in experimental data to infer instrument calibration, such as MSQuant [52], DtaRefinery [53], and nonparametric regression model [54]. In addition, Preview [55] can apply a fast database search to evaluate precursor and fragment mass error and nonspecific digestion during the experiment. Param-Medic [47] is a search parameter inference tool designed to determine the optimal search parameters by assembling and analyzing pairs of spectra that are likely from the same peptide ion to infer precursor and fragment mass error. Unlike Preview, Param-Medic is implemented in Python as a standalone tool and is convenient to be integrated into the stream pipeline for Linux users.

The scoring function of PSMs also plays an important role in the database searching approach. A well-calibrated scoring system is necessary for distinguishing among the different spectra. For example, Mascot uses probability-based scoring in which the total score is related to the probability that an observed match is a random event. SEQUEST [56] applies two scoring functions: the first one is used to select a limited range of peptide candidates for each spectrum (Sp), and the second uses cross-correlation between the observed and theoretical spectra (Xcorr). The recent version of MaxQuant software has incorporated the Andromeda search engine. Andromeda uses a score based on the probability of matching at least k out of the n theoretical masses by chance from the filtered MS/MS spectrum, where n is the total number of theoretical ions and k is the number of matching ions. According to the evaluation from Cox et al. [57], the score functions used by MaxQuant and Andromeda have very similar discriminatory power, while Andromeda achieves better accuracy in highly phosphorylated peptides. The support vector machines (SVM)-based peptide statistical scoring method is an effective way to reduce the false discovery rate (FDR) in peptide identification [58]. The presence of the candidate peptide can then be determined by the SVM model trained with the vector representations of peptides from different database search metrics. Lin et al. [59] proposed the “residue evidence” score and demonstrated that this score function can result in performance improvements in a variety of datasets without introducing new trainable parameters. Another application, called PepHMM [22], introduced a new scoring function to improve the accuracy of peptide identification by integrating information on raw data accuracy, peak intensity, and correlation among known and detected peptides into a hidden Markov model to generate a confidence score based on statistical significance.

The database search approach is often followed by a second round of searching against a decoy database in order to reduce FDRs [60,61]. This procedure ensures that all remaining peptides have an FDR higher than the predefined cutoff for peptides identified from the decoy database. Decoy database searching estimates a threshold for removing identifications that have low statistical confidence, resulting in a higher percentage of true positive hits. Several commonly used protein sequence databases for spectrum matching are listed in Table 1. Many widely used analysis platforms, such as MASCOT [17] and MaxQuant [18], have integrated this strategy in their standard analysis pipelines. However, with the increasing size of the protein sequences database, the target-decoy search strategy is becoming computationally inefficient since its searching space is twice as large as the original target database search. To resolve this problem, many novel searching strategies have been reported in recent years. Gonnelli et al. [46] proposed Nokoi, which is a decoy-free approach for improved peptide identification accuracy. This approach is based on an L1-regularized logistic regression model trained on a large heterogeneous dataset using ranks supplied by the Mascot as true labels for PSMs. Kim et al. developed a target-small decoy search strategy that can handle cases where the sizes of target and decoy databases differ, which is done by reversing the target database and picking random proteins according to the ratio of small-decoy to target database sizes. Their evaluation shows that this approach reduces the database size and searching time while retaining the same level of accuracy as normal target-decoy search [62].

In de novo peptide sequencing, the peptide sequence is determined solely from fragmentation spectra information and fragmentation method properties. The analysis strategy for de novo peptide sequencing has been dominated by Graphical Probabilistic Model and Hidden Markov Model (HMM)-based methods, such as PepNovo [14] and NovoHMM [29,63]. However, many variants of the sequencing frameworks have been reported since then. In order to solve the performance deterioration in Electron Transfer Dissociation spectra and Higher-energy Collisional Dissociation spectra, Jeong et al. proposed a universal de novo peptide sequencing algorithm, called UniNovo [34]. UniNovo incorporates a new scoring criterion calculated from a modified offset frequency function to capture the dependencies between different types of ions. Low ion coverage is another well-known challenge in de novo peptide sequencing since the order of consecutive amino acids cannot be easily determined if all of their supporting fragment ions are missing. To address this problem, Yang et al. [32] recently proposed pNovo 3, which specifically designed to distinguish peptide candidates of each spectrum with a learning-to-rank framework. pNovo 3 can also generate different metrics measuring the similarity of the actual experimental spectrum and the theoretical spectra predicted by deep learning. Other deep learning-based methods [27,64] have been introduced to solve highly multiplexed spectra in recent years. These models often incorporate the highly customized architecture of convolutional and recurrent neural networks and can be trained with features attainable for de novo sequencing, such as spectra data, fragment ions information, and sequence patterns of amino acid.

It is also possible to achieve better performance by combining de novo peptide sequencing with the database matching approach. Such hybrid approaches first choose the most suitable protein database for searching based on peptide tag sequences and then perform error-tolerant searching against the selected database. This category of searching strategy includes InsPecT [37], DirecTag [36] and JUMP [38]. In contrast, PEAKS Studio [39] performs de novo sequencing of spectra before any database searching. More recently, Cifani et al. [40] proposed ProteomeGenerator, which is a hybrid proteomics framework. This new approach calibrates the matching results from a target–decoy database with sample-specific controls, and can significantly improve the accuracy of isoform identification in non-canonical proteomes. Parallel PSM processing algorithms for large scale proteomics dataset have also been implemented [33].

Data-independent acquisition (DIA) mass spectrometry is now emerging as a new strategy for systematic analysis of peptide mixtures. Unlike traditional data-dependent acquisition (DDA), in which mixtures of precursor ions are selected based on co-selection and co-dissociation, DIA detects all fragmented ions at each chromatographic time point within a predefined m/z range, or uses the m/z ranges during isolation and fragmentation [65]. The strength of DIA is that all precursor ions are selected without bias, providing more reliable input for downstream systematic analysis. Various analysis strategies have been developed for DIA approach with different approaches. For example, MSPLIT-DIA [19] is a library matching method in which input from DIA spectrum are searched against library spectra and spectrum projections are evaluated based on normalized dot product. However, results from these library-based methods are limited to prior knowledge from data present in the library. To address this issue, many library-free methods have also been proposed. These tools typically reconstruct pseudo spectra from deconvolving the multiplexed spectra or highly correlated precursor ion groups, such as DIA-Umpire [42], or directly compute the confidence of existence for each query peptide from DIA data, such as FT-ARM [66] and PECAN [31]. Recently, Searle et al. [67] proposed a rapid, experiment-specific library generation workflow for DIA-MS for non-model organisms and non-canonical databases. In their system, libraries containing every peptide in a proteome are constructed first, followed by a refinement process using empirical data built directly from protein sequence databases.

Posttranslational modifications (PTMs) in proteins can also be identified with MS. However, simply searching the database with all possible modifications positions can be extremely time-consuming since the large number of all possible position/modification combinations. Several methods exist to help decrease the computational requirements or increase the accuracy of results, such as ModifiComb [45], PTMselect [51], and G-PTM-D [68]. PEAKS PTM [69] is the PTM identification method integrated in the PEAKS Studio software. In PEAKS PTM, unassigned spectra with high de novo scores are searched against the identified proteins. Sequence data from genomic or transcriptomic databases can also aid peptide identification by providing possible protein sequences. This strategy is widely used in proteogenomics, which is an emerging area of research that uses information from both genomic and transcriptomic databases to facilitate the identification of novel peptides from MS-based proteomics data [70]. MetaMorpheus [50] is another novel tool, which incorporating multinotch searches in global PTM discovery. Compared with the G-PTM-D approach, it achieves a higher number of identified PTMs with a significant increase in search speed.

Once the peptide identification is complete, the next step is to reconstruct the peptide sequences into original proteins. This procedure is called protein inference. Longer peptides are more informative in this step due to their uniqueness. In comparison, it is usually not straightforward to construct a reliable list of proteins from shorter peptides, since some of the peptides may be shared by two or more proteins. These “degenerate peptides” usually have multiple optimal solutions for protein assignment. Many models built for peptide assembly adapt the parsimonious rule, in which the smallest set of proteins that account for the detected peptides is reported [41,43,71]. Probabilistic models are also widely used in protein inference and was first introduced by ProteinProphet [72]. More statistical modeling frameworks, such as Hierarchical Statistical Model [73] and Bayesian Inference Model [74], have been proposed since then. The Bayesian Inference Model applies Bayesian models built based on posterior probabilities and has improved the performance of the original ProteinProphet by approximately 6%. The performance of protein inference is often affected by the strength of evidence, such as the PSM score from the observed peptides [75]. Typically, for large proteomics data sets, a stringent cutoff for protein identification FDRs is set to reduce the number of incorrectly reported proteins. Novel FDR estimation frameworks for protein inference have also been developed. Reiter et al. proposed MAYU [44], which extends the target-decoy strategy for FDR estimation of PSM to the protein level. Validation in various large proteomics datasets shows that the size of the dataset impacts the reliability of protein identifications results. Recently, Wu et al. [76] proposed a new FDR estimation approach in which the null distribution is generated from a combination of logistic regression model with the Permutation + BH (Benjamini–Hochberg) method. Moreover, the authors showed that this approach achieves consistently better performance than MAYU.

### 2.2. Protein Abundance Quantification

The abundance level of proteins across the entire proteome in a sample can be acquired from quantitative proteomics analysis. The increasing volume and extent of quantitative proteomics data inspired the development of numerous subsequent bioinformatics analysis methods. Currently, there is no standard analysis pipeline to construct the global protein expression profiles from samples in a straightforward fashion, since many software and algorithms have been developed for dedicated upstream experimental techniques. Nevertheless, most experimental quantification methods fall into two categories: labeled methods and label-free methods. In this section, we first review the latest software tools designed for both techniques, and then introduce several software tools dedicated for an emerging technique in clinical studies, called imaging mass spectrometry. Table 2 lists software tools for protein abundance quantification reviewed in this section.

Currently, there are two popular approaches for label-based quantification: MS1 (first stage MS) based labeling [88,89], and MS2 (second stage MS) based labeling. In MS1 based labeling, different samples will have different isotope patterns in MS1 spectra depending on their labeling. This can be done for both in vitro and in vivo samples. Isotope coded affinity tags (ICATs) [90], dimethyl-based labeling [91] and isotope coded protein labeling (ICPL) [91] are common strategies for in vitro experiments. Stable isotope labeling by amino acids in cell culture (SILAC) [92] and ^15^N metabolic labeling [93] are the most popular strategies for in vivo labeling. Various bioinformatics methods have been developed for MS1 labeling experiments, including MaxQuant [18], PVIEW [80] and XPRESS [81]. Most software tools can handle samples from multiple labeling methods. For example, XPRESS is able to analyze ICAT, SILAC, and ICPL labeled samples, and it can also calculate the relative abundance of proteins based on the elution profiles of the labeled peptide pairs. PVIEW can handle SILAC, ICPL and ICAT labeled samples, and it can even perform nonlinear alignment label-free quantification and label free XIC-based quantification. On the contrary, MaxQuant is designed specifically for high-resolution data with SILAC labeling from Thermo Orbitrap and FT mass spectrometers. With the Perseus framework [48], it is convenient to perform downstream statistical analysis for raw quantification results from MaxQuant.

**Figure 2:**
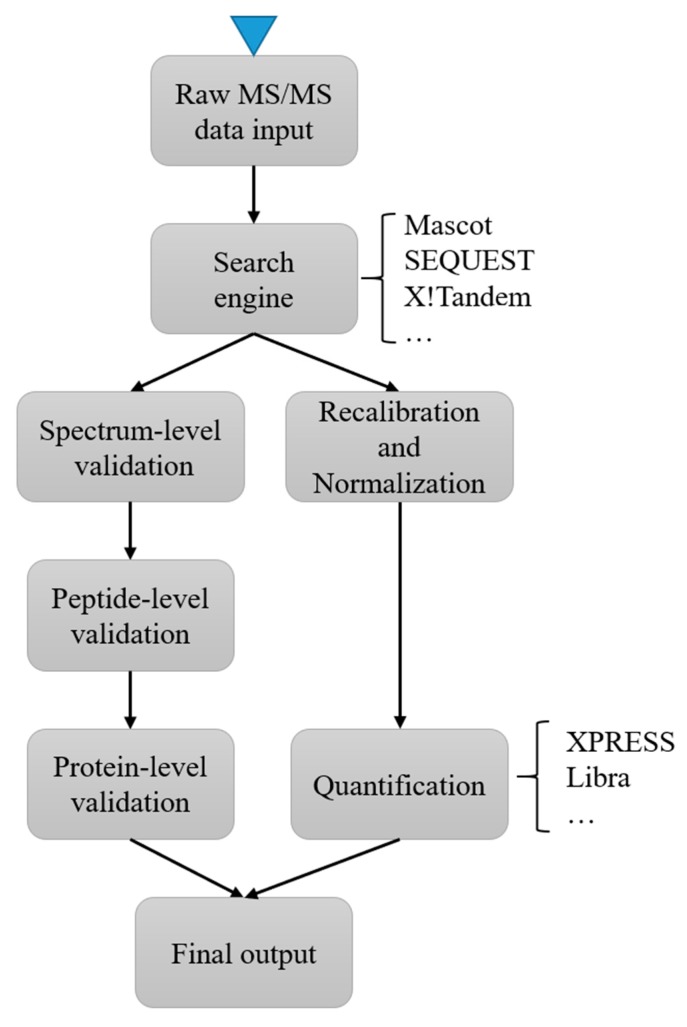
A typical workflow for protein abundance quantification using Trans-Proteomic Pipeline (TPP). After the raw data files are converted to one of the supported open XML formats, the data are processed with a designated search engine and the search results are validated at spectrum, peptide, and protein level. Meanwhile, the quantification tools will perform the protein quantification with the normalized data. The final output are ready for various downstream analysis.

In MS2 based labeling, the quantification signals can be detected at the low mass range of MS2. Isobaric labeling is a common strategy in MS2 based labeling [94]. In isobaric labeling, samples are labeled with certain chemical groups (such as TMT [95] and iTRAQ [96]) that have identical mass but a different distribution of heavy isotopes. It can achieve relatively large multiplexing capacity during an MS run but has the problem of ratio compression due to cofragmentation [97]. Most instrument vendors provide commercial software for the analysis of raw data from spectrometers. For example, the ProteinPilot (from ABSciex) and the Proteome Discoverer (from Thermo Scientific) can handle raw data from their corresponding instrument vendors. There is also a wide range of free software available, including iTracker [78], IsobariQ [77] and Libra [98]. These free software tools often use public peak file formats, such as mzXML, to import and process data. In addition, the Trans-Proteomic Pipeline (TPP) [79], provides a full workflow from raw data processing to statistical analysis. TPP integrated a collection of software used in quantification analysis, such as XPRESS and Libra, and can process isotopically or isobarically labeled samples as a single software system. Figure 2 illustrates a typical workflow of protein abundance quantification using TPP. Skyline [87] and OpenMS [85] are similar integrated platforms for convenient and flexible analysis of MS data.

In label-free quantification, spectra for different samples are acquired from separate LC-MS/MS experiments [99]. However, this may introduce unwanted variations, such as the inconsistency of chromatographic measurements from multiple experiments. To address this issue, different intensity normalization methods have been implemented, such as total ion count (TIC), MaxLFQ (integrated into MaxQuant) [83]. Usually, the label-free quantification is performed based on analysis of the signal intensity of peptide precursor ions of the fragmented proteins, and most software are designed using this approach, such as Mascot Distiller [100], MaxQuant [18], and VIPER [84]. In addition, tandem mass spectra counting can be used as an alternative quantification approach. The exponentially modified protein abundance index (emPAI) [82] integrated into Mascot is one of the tools using this approach. The observed peptide count can also be corrected by the peptide detection probability based on characteristic physicochemical peptide properties using machine learning models [101].

Models such as iBAQ [102] and LCMSE [103] are built to measure the global absolute quantifications of proteins. For example, iBAQ values are generated from dividing total intensities by the identified peptides for one protein. Nonetheless, it is infeasible to directly compare the abundance levels of different proteins due to their distinct efficiencies of ionization and detectability. Spike-in standards [104] or the “proteomic ruler” approach [105] can be applied to improve the quality of absolute abundance estimation.

Imaging mass spectrometry (IMS) is emerging as a popular protein quantification technique in clinical studies, particularly in neuroscience [106]. IMS has the capability to systemically detect localization patterns of proteins and their abundance in various types of cells and biological tissues. In IMS, proteins from different biological samples are desorbed and ionized with different probes. Popular techniques used in IMS include laser desorption/ionization (LDI), matrix assisted-LDI (MALDI) [107], time-of-flight -secondary ion mass spectrometry (TOF-SIMS) [108] and electrospray based desorption (DESI) [109]. These techniques are designed for various purposes with respect to their different capability of spatial resolution and detection power. In order to improve the quality of raw images from IMS, image processing algorithms such as edge-preserving image denoising/clustering [110] and spatially aware clustering [111] have been proposed to generate more informative and precise segmentation maps. Software suites that can perform baseline corrections, subtractions, denoising, smoothing, recalibration, and normalization of IMS data in an integrated pipeline have also been developed [112].

## 3. Data Preparation and Downstream Bioinformatics Analysis in Proteomics

### 3.1. Data Preprocessing and Normalization

In peptide-based MS quantitation, the abundance of a protein is inferred from a limited number of peptides, sometimes only one. Typically, researchers need to carefully apply cutoffs for minimum peptide numbers to increase the reliability of the inferences from only a small number of peptides. For instance, Peaks Studio Quantification uses only the top three peptides for the quantification of a protein, while it should be using as many as possible as long as they are reliably detected. In addition, to generate comparable and reliable results for downstream analysis, it is always necessary to perform preprocessing and normalization of the raw quantitative data.

Normalization refers to a data correction process that removes any non-biological related variations and makes the results reliable and aligned across samples for downstream analysis. Selecting an appropriate normalization method is crucial in proteomics analysis, especially considering the fact that inadequate normalization is usually being performed prior to LC/MS [113]. Several types of normalization methods are developed based on different statistical assumptions. The distribution of the protein abundance is often extremely skewed towards zero, so it is common practice to perform logarithm transformation on the intensity values before further normalization. For linear regression-based normalization such as RlrMA and LinRegMA [114], they assume that bias in the measurements and the scale of the observed protein abundance are linearly dependent. For local regression normalization [115], the assumption is that bias and protein abundance have a nonlinear dependency and can be explained with nonlinear models such as local polynomial regression. Variance stabilization normalization (VSN) [116] is another statistical model, which aims to eliminate the dependency between variances and mean abundances and scaling data from different samples into the same level through parametric transformations and maximum likelihood estimation. Quantile, median and EigenMS [117] normalizations are also used in proteomics in various studies. Välikangas et al. [114] systematically evaluated eleven commonly used normalization techniques in four separate proteomics datasets, and the results indicate that these normalization methods generally have the same level of performance on most of the proteomics data, while VSN generally performed consistently well in the differential expression analysis.

Despite the various normalization methods mentioned in this section, the optimum normalization approach is dependent upon the experiment environment in different MS-based proteomics studies and needs to be evaluated individually with MA plots. Moreover, hierarchical clustering and heatmaps are useful visualization techniques to help to determine the effectiveness of the preprocessing steps.

The raw quantitative data may also contain missing values, especially for those with relatively low concentration in the cellular context. Missing values are common in most high throughput technologies due to the stochasticity in sampling during the experiment [118]. These missing values can be removed or included based on the distribution of all proteins detected. Various machine learning models have been developed to impute missing values for omics data, including empirical distribution sampling [119], k-nearest neighbors (kNN) imputation [120] and singular value decomposition (SVD) imputation [121]. However, it has been shown that the performance of these imputation methods will decrease drastically when the data contains more missing values [122]. Novel missing value approaches have been developed with higher imputation accuracy and statistical sensitivity. For example, Wei et al. [123] proposed GSimp, which adapts an iterative Gibbs sampler to infer the missing values for MS data. More recently, Berg et al. [119] developed an efficient imputation model based on sampling from normal distributions with better performance at high false positive rates level.

### 3.2. Statistical Analysis of Quantitative Protein Data

Once the protein abundance data has gone through data cleaning, filtering, and normalization process, it is ready for further statistical analysis and further downstream studies. The most straightforward statistical analysis is to examine if there are any significant changes in protein levels between two different conditions. This is typically done by performing a t-test between the observed protein abundances from two different groups [124]. In case there are two or more factors required to estimate, ANOVA (analysis of variance) [125] can be performed instead. However, the sample size in proteomics data is relatively small due to the limited multiplexity, and this impairs the statistical power of the t-test and results in insignificant *p*-values for proteins with a large fold change, but also a relatively large variance. To address this issue, several statistical models have been proposed. For example, Kammers et al. [126] demonstrated better results can be achieved by applying moderated t-statistics from the empirical Bayes procedure Linear Models for Microarray Data (LIMMA) in the identification of differentially expressed proteins. The LIMMA model, as is suggested by its name, is originated from significant change detection in microarray data. LIMMA is designed to reduce the variances of the measurement to a pooled estimate based on all sample data and can achieve more robust and accurate results than traditional t-test, especially on relatively small proteomic datasets. Linear mixed-effects models [127], mean/median sweeps [128], and “masterpool” normalization [128] are other commonly used methods in relative protein abundance estimation and statistical analysis of proteomic data.

For all statistical tests, it is necessary to set up an FDR threshold since multiple hypotheses are being tested at the same time. The Benjamini–Hochberg procedure [129] and FDR estimation from permutation [130] are widely used approaches in proteomics. It has been demonstrated that introducing FDR-controlling procedures can effectively reduce the number of false positives in the statistical analysis of proteomic data [131].

### 3.3. Enrichment Analysis in Proteomics

Enrichment analysis is a method that helps to identify genes or proteins that are overrepresented in the predefined gene set of interest. The gene set usually consists of a list of genes that share common functions or in the same pathway or network. The advantage of performing enrichment analysis on proteomics data is that we can test hypotheses on the systemic measurements of the proteome instead of the transcriptome. Moreover, the input of such analysis can incorporate additional information after the transcription process, such as differential translation rate and PTM. Publicly available online databases, such as DAVID [132] and STRING [133], have provided gene sets built from prior knowledge and automatic tools to perform automatic enrichment analysis online. For PTMs, databases such as PhosphoSitePlus [134] and Signor [135], have curated additional information about modification position/type and their related diseases from literature mining. In such workflows, the users provide a list of gene identifiers or modifications and then select a gene set database based on their research interest. While databases such as Uniprot and Ensembl can accept protein names as input, many other databases require converting protein names into gene names before further steps. However, it is still a challenging task for identifier conversions as protein names and corresponding genes are in a many-to-many relationship, and such conversions are often labor-intensive and will result in information loss. Currently, several web cross-reference services are developed to solve the conversion tasks, such as PICR [136] or CRONOS [137], but the users should be cautious since these tools may not represent up-to-date information and may need manual correction. It is also worth mentioning that biological databases are rapidly growing in size and number, whereas accurate curations of these new data are often lagging (the “Data avalanche”) [138]. Inadequate database curation and a lack of common data formats for cross-database referencing often results in labor-intensive work before enrichment studies.

Gene Ontology (GO) Enrichment [139] is the most widely used technique in enrichment analysis. The GO terms can be considered as a set of predefined groups to which different genes are assigned based on their functional characteristics, thus helping to reduce the redundancy in terminologies. The GO terms are hierarchically clustered with the top nodes describe the three main categories of the terms: “biological process”, “molecular function”, and “cellular component”. Each term has a unique identifier and is connected to other related terms. The Amigo database [140] provides GO term annotation for many species, but not all proteins have a complete and precise annotation. For those proteins that do not have complete annotations, informative GO terms from proteins with similar sequences can be used instead. GO term prediction algorithms such as ProLoc-GO [141], PFP [142] and IGNA [143] are designed for this problem. The most common statistical tests used in GO enrichment are Fisher’s exact test and the hypergeometric test. Statistically significant GO terms are those that appear more frequently in the input protein list than would be expected by chance, and they may indicate interesting biological processes for further studies. However, since GO terms usually represent ORF products, rather than mature proteoforms, researchers should carefully examine the GO terms in the enrichment results to ensure that they have appropriate relations between the proteoforms and corresponding genes.

**Figure 3:**
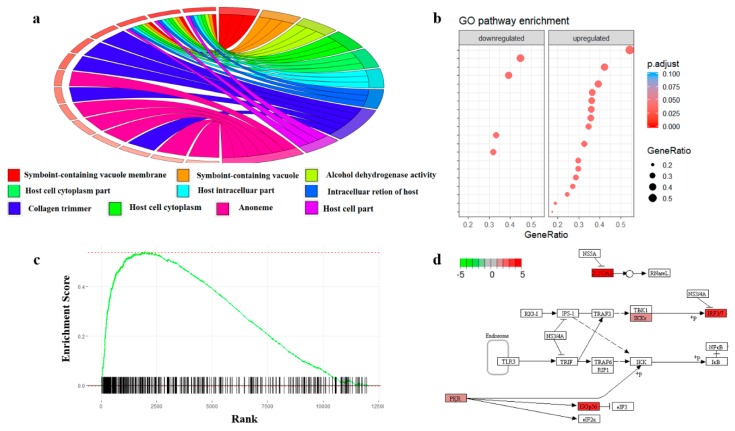
Illustration of enrichment analysis with proteomics data. (**a**) Gene Ontology (GO) enrichment with Circos plot. The left part of the circle shows the differentially expressed proteins and their significant levels. The right part shows the enriched GO Terms. (**b**) Pathway enrichment in dot plot. The colors of the dots represent the adjusted *p*-value and the sizes of the nodes represents the ratio of the proteins that are differentially expressed to the total proteins in the pathway. (**c**) The running sum score of the gene set enrichment analysis (GSEA). Significant proteins will increase the enrichment score and proteins that are not in the list will decrease the score. (**d**) Protein enrichment with KEGG pathway database with Pathview. The color of the boxes represents the log2 fold change of the protein abundances.

Similar to GO terms, prior knowledge about regulatory pathway networks and diseases can also be used to perform enrichment analysis [144]. A biological pathway describes biological actions and chemical reactions among molecules within a cell that result in certain biological processes. Databases such as PANTHER [145] and Reactome [146] curated the interaction maps for different pathways as well as a set of enrichment tools to perform enrichment directly on the database webpage. Many independent tools can also use the public interface to run enrichment analysis based on the data curated in those only databases directly. For example, Pathview [147] is an R package for KEGG pathway [148]-based data integration and visualization. Protein set enrichment analysis (PSEA) is another popular enrichment approach and is derived from gene set enrichment analysis (GSEA). In PSEA, the enrichment score is computed from a weighted running sum statistic and proteins without significant changes in abundance may negatively affect the enrichment score. While many GSEA software can also perform PSEA, tools developed specifically for protein quantification data, such as PSEA-Quant [149], may provide a more convenient and reliable workflow for proteomics. Figure 3 shows the common enrichment analysis visualization tools in proteomics.

### 3.4. Machine Learning Approaches

Machine learning is powerful in downstream bioinformatics analysis. It can extract informative features from a huge amount of proteomics data and construct models that can be validated from separate datasets rather than providing simple descriptive information. There are two main types of tasks of machine learning: supervised learning and unsupervised learning. Common applications with supervised learning often involve using the quantitative proteomics data to build models that can predict the annotations for new samples. The format of the labels in supervised learning can be either discrete categories (classification) or continuous numeric values (regression). For example, clinical outcomes (such as survival time) can be predicted from quantitative proteomics data with models trained from samples collected from patients with known clinical information [150]. Applications of supervised learning in proteomics usually involve taking the quantitative results from MS as input and manually labeled or experimentally verified annotations as the target output. In unsupervised learning, the goal is to infer the natural structure and dependency existed within the data given. Both supervised learning and unsupervised learning are powerful methods in the downstream analysis for proteomics, and enormous tools have been implemented for various research purposes.

Common models in supervised learning include Bayesian classifiers, Logistic Regression, Decision trees, Random Forest, Support Vector Machines (SVM), and Artificial Neural Networks. A variety of applications in proteomics is reported in several works. Deeb et al. [151] used protein expression profiles to perform classification for patients with diffuse large B-cell lymphoma. According to their study, highly ranked proteins from the trained models can be recognized as core signaling molecules in pathobiology for different subtypes. Dan et al. [152] used an SVM classifier to identify diagnostic markers for tuberculosis by proteomic fingerprinting of serum. Their model also helped to identify several potential biomarkers for new diagnostic methods. Tyanova et al. [153] used proteomic profiles to identify functional differences between breast cancer subtypes. They further identified several proteins with different expression signatures across the subtypes without the involvement of gene copy number variations. These findings provide novel insight into future subtype-specific therapeutics development. Protein subcellular localization is another fruitful field for supervised learning methods [154]. Due to the nature of high dimensionality for proteomics data, dimensionality reduction techniques such as principal component analysis (PCA), Linear Discriminant Analysis (LDA) and t-Distributed Stochastic Neighbor Embedding (t-SNE) are often applied prior to building the models [155]. Regularization is another widely used technique in supervised learning. It reduces the model complexity and number of features required for prediction by adding larger penalties to models that have more parameters. Deep Learning also becomes a popular approach in proteomics since it is good at extracting useful information from large numbers of features. For example, Ding et al. constructed a deep autoencoder model to identify informative features from genomics and proteomics data, which was then used to predict the effectiveness of drugs in cancer cell lines [156]. They further showed that the features derived from deep learning models contain relevant information of the cellular signaling system and contribute to the improvements of model accuracy. Cross-validation is widely used for performance assessment in supervised learning [157]. It also helps to avoid the overfitting problem, since the performances of the models are always evaluated from separated validation datasets rather than the samples used in model training.

The simplest form of unsupervised learning is hierarchical clustering, according to protein abundance values. Clustering can be used as a quality assessment for MS experiment results as we can compare the blindly grouped samples with the prior knowledge about sample similarities. Several more complicated unsupervised learning approaches have been reported: Peptide identification Arbiter by Machine Learning (PepArML) [158] is an unsupervised learning method built to determine the peptide associated with the tandem MS spectrum. ProtVec [159] is an unsupervised distributed representation for biological sequences and can be applied to many common problems in proteomics, such as protein family classification and structure prediction. Compared with supervised learning, unsupervised learning is less frequently used in proteomics. However, it does not require ground truth for model training and is particularly useful in heavy data-driven tasks such as feature extraction.

## 4. Protein Networks Reconstruction

In biomedical studies, a new trend is to explore the complex contextual relationships between molecules of interest within the cellular environment. This paradigm created a new branch in life sciences, called systems biology or networks biology [160]. Unlike the traditional mechanism studies, which follow the “one gene, one protein, one function” principle, the systems biology views the interactions between genes and their functions as a large network. In this network, the nodes represent functional molecules within cells, such as genes or proteins. The edges between nodes indicate the functional relationship between the molecules. The connection can be either direct interactions such as chemical reactions between kinases and their substrates, or indirect relationships such as the transcriptional regulation between transcription factors and their targets. Analyzing the biological processes with the networks graphical model provides a novel perspective between genotypes and phenotypes and can also efficiently utilize the huge volume of omics data.

Currently, there are two main categories in proteomics-based network biology: protein-protein interaction (PPI) networks and signaling networks. The PPI network describes the direct interactions between two proteins and can be validated through the analysis of protein complexes using affinity purification-mass spectrometry (AP-MS) [161]. In AP-MS, certain proteins of interest are tagged and then detected from copurified protein components by MS. However, false-positive interactions can also be introduced during the experiment. Several computational frameworks have been established to compensate for limitations in experimental design and a lack of proper controls in AP-MS. Rinner et al. [162] proposed the MasterMap system that facilitates the quantitative protein complexes analysis with label-free MS. Glatter et al. [163] proposed the PP2A integrated workflow that significantly improved the data throughput, sensitivity, and robustness in human protein-based global AP-MS analysis. Many graph-based clustering methods, such as superparamagnetic clustering [164] and hyperclique pattern discovery [165] also proved to be effective approaches for detecting actual functional protein complexes.

An objective of studying signaling networks is to interpret the relationships between enzymes and their substrates. The interactions between kinases and their targets are particularly interesting since they are essential cellular signaling molecules and frequently associated with diseases such as cancers and endocrine disorders [166,167]. MS-based phosphoproteome analysis of kinase signaling also helps reveal the regulatory modification pattern of proteins in bacteria [168,169,170]. Moreover, proteomics can help identify the acetylation modification, which has emerged as a major interest in epigenetics and cellular signaling recently [171,172,173,174]. With the advancements in MS-based techniques, it is now possible to identify a wide range of PTM events, such as phosphorylation in a single MS run with high coverage and quality. Furthermore, the PTM information can help to predict the status of the nodes and edges in signaling networks. One important application in this field is kinase activity inferences based on abundances of phosphorylated proteins. Several machine learning methods, such as IKAP [175], KSEA [176], and kinact [177], have been implemented for this task. Reconstruction of the signaling network is another important topic. In the HPN-DREAM challenge held by Dialogue for Reverse Engineering Assessment and Methods (DREAM), one of the main categories is to infer causal signaling networks from time-course Breast cancer proteomic data. A systematic assessment [178] has been published with a comprehensive evaluation of all submitted models. Since then, many new network inference algorithms have been developed, such as PerseusNet [48], GNET2 [179], Neglog [180] and HIPPIE 2.0 [181]. Dynamic network reconstruction models for time-series proteomics data have also been reported. Usually, they are designed to quantify changes in network in response to different physiological conditions or when stimuli factors are introduced. COVAIN [182] is an integrated toolbox using time-series and correlation network analysis to investigate responses on different hierarchies of cellular contexts. Considering the popularity of network studies in biology, most network inference tools have been packaged as R and Python libraries, or integrated web services for better accessibility among the academic community.

Tremendous effort has been made to elucidate disease pathogenesis in a network view. Wang et al. [183] showed that Se-and Zn-related proteins play significant roles in Endemic Dilated Cardiomyopathy Keshan Disease from networks constructed from protein expression profile. Pirhaji et al. [184] designed a random forest-based algorithm called PIUMet, to study abnormal signaling pathways in metabolisms of sphingolipids, fatty acids, and steroids within Huntington’s disease. Many biological network databases are freely available to the public, such as KEGG [148], Pathway Commons [185] and BioGRID [186]. Usually, these databases will also provide programmatic interfaces that allow bioinformaticians to run comprehensive analysis without the need to understand the structure and format of data stored. However, it is still a challenging task to systematically infer the protein networks since most species have a large pool of proteins. Thus, network inference problems usually have an extremely high computation cost and are often limited by available computational resources.

## 5. Discussion and Future Perspectives

MS-based proteomics has greatly improved our understanding of the complex biological mechanisms that underlie human health and disease. Currently, the upstream of MS-based proteomic analysis, including protein identification, characterization, and quantification, is becoming increasingly convenient and reliable as automated pipelines have been provided in most of the experimental platforms. New multiplexing technologies have made it possible to analyze hundreds of samples at a higher throughput. With the latest isobaric tag-based multiplexing, up to 11 samples can be analyzed in a single MS run [187]. Despite recent advancements, several major issues still exist for proteomics. Similar to next-generation sequencing, it is still challenging to precisely quantify proteins at low abundance level. Moreover, reducing the number of missing values is not always possible. Novel techniques that can improve the effectiveness of ion sources, spectra resolution, and dynamic detectors with broader range may become the trend for future upstream proteomics development.

Combining the proteome data with other omics technologies is emerging as a new paradigm in bioinformatics. For example, pointwise comparisons between proteomics and transcriptomics can be established since the identifiers of genes and proteins can be mapped between the two omics spaces. Proteins/genes with discordant trends may indicate the involvement of significant transcriptional and post-translational regulation mechanisms. Quantitative proteomics using in vivo SILAC mouse technology has been successfully applied in studying the post-transcriptional mechanisms in circadian regulation of the liver [188]. The reverse engineering of protein signaling networks can also benefit from other omics data. Virtual Inference of Protein-activity by Enriched Regulon (VIPER) algorithm [189] can perform computational analysis of protein activity based on the relationship between transcription factors and their potential targets identified from transcriptome data. Given the fact that different types of omics data are often complementary to each other, the MS-based proteomics will become much more powerful when analyzed from a multi-omics perspective.

As MS-based proteomics technology continues to evolve, the associated bioinformatics tools need to be updated accordingly. So far, most of the analysis methods mentioned in this review have been provided with a convenient and user-friendly interface. Table 3 lists all downstream bioinformatics tools and databases for proteomics analysis mentioned in this review. We expect more bioinformatics methods from interdisciplinary studies will emerge in the future and enhance the current understandings of complex systems biology.

## Figures and Tables

**Table 1:** Commonly used software packages for peptide and protein identification.

Category	Name	Description
Database search algorithms	Andromeda [18]	Probabilistic scoring-based peptide search engine integrated in MaxQuant.
	Mascot [17]	Probability-based database searching algorithm.
	MSPLIT-DIA [19]	Sensitive Peptide Identification for Data Independent Acquisition.
	MudPIT [20]	Multidimensional protein identification.
	PepArML [21]	An Unsupervised, Model-Free, Machine-Learning Combiner for Peptide Identifications from Tandem Mass Spectra.
	PepHMM [22]	A hidden Markov model-based scoring function for mass spectrometry database search.
	Protein Prospector [23]	An integrated framework of about twenty proteomic analysis tools.
	SEQUEST [24]	An approach to correlate tandem mass spectral data of peptides with amino acid sequences in a protein database
	TopPIC [25]	A software tool for top-down mass spectrometry-based complex proteoforms identification.
	X!Tandem/X!!Tandem [12,26]	An open source software that search tandem mass spectra with peptide sequences in database.
De novo peptide sequencing	DeepNovo-DIA [27]	De novo peptide sequencing by deep learning.
	EigenMS [28]	De novo Analysis of Peptide Tandem Mass Spectra.
	NovoHMM [29]	A hidden Markov model for de novo peptide sequencing.
	PEAKS [30]	A fast de novo sequencing tool.
	PECAN [31]	Library Free Peptide Detection for Data-Independent Acquisition Tandem Mass Spectrometry Data.
	PepNovo [14]	De novo peptide sequencing via probabilistic network modeling.
	pNovo 3 [32]	A software for precise de novo peptide sequencing using a learning-to-rank framework.
	SHERENGA [13]	De novo peptide sequencing via tandem mass spectrometry.
	SWPepNovo [33]	An Efficient De Novo Peptide Sequencing Tool for Large-scale MS/MS Spectra Analysis.
	UniNovo [34]	A universal de novo peptide sequencing algorithm with a modified offset frequency function.
Hybrid identification approach	ByOnic [35]	A hybrid of de novo sequencing and database search for protein identification by tandem mass spectrometry.
	DirecTag [36]	Accurate sequence tags from peptide MS/MS with statistical scoring method.
	InsPecT [37]	A software for identification of peptides posttranslational modification (PTM) from tandem mass spectra.
	JUMP [38]	A tag-based database search tool for peptide identification.
	PEAKS DB [39]	A hybrid de novo sequencing tool run in parallel with database search.
	ProteomeGenerator [40]	A hybrid framework for based on de novo transcriptome assembly and database matching.
Other software related to protein/PTM identification	DBParser [41]	Web-based software for shotgun proteomic data analyses.
	DIA-Umpire [42]	Comprehensive computational framework for data independent acquisition proteomics.
	MassSieve [43]	Panning MS/MS peptide data for proteins.
	MAYU [44]	A novel strategy that reliably estimates false discovery rates for protein identifications in large-scale datasets.
	ModifiComb [45]	Mapping substoichiometric post-translational modifications.
	Nokoi [46]	A decoy-free approach for improved peptide identification accuracy
	Param-Medic [47]	A strategy for inferring optimal search parameters for shotgun proteomics analysis.
	Perseus [48]	Platform for comprehensive analysis of proteomics data.
	PROVALT [49]	A heuristic method for computing false discovery rate (FDR) for protein identifications.
	MetaMorpheus [50]	Enhanced Global Post-translational Modification Discovery.
	PTMselect [51]	Optimization of protein modifications discovery by mass spectrometry.

**Table 2:** Commonly used software package for quantitative proteomics.

Category	Name	Description
Label-based	IsobariQ [77]	A relative quantification software that can be used for both iTRAQ (Isobaric tags for relative and absolute quantitation) and TMT (Tandem mass tag) labeling.
	iTracker [78]	Allows quantitative information gained using the iTRAQ protocol to be linked with peptide identifications from popular tandem MS identification tools
	Libra [79]	The iTRAQ quantification module of the TPP (Trans-Proteomic Pipeline).
	MaxQuant [18]	One of the most frequently used platforms for mass-spectrometry (MS)-based proteomics data analysis.
	ProteinPilot (ABSciex)	Full solution for protein identification and label-based protein expression experiments.
	Proteome Discoverer (Thermo Scientific)	Proteomics workflows for a wide range of applications.
	PVIEW [80]	LC-MS/MS Data Viewer and Analyzer developed by Princeton.
	XPRESS [81]	Quantitative profiling of differentiation-induced microsomal proteins using isotope-coded affinity tags and mass spectrometry.
Label-free	emPAI [82]	Exponentially modified protein abundance index.
	Mascot Distiller (Matrix Science)	A single, intuitive interface to a wide range of native (binary) mass spectrometry data files.
	MaxLFQ [83]	Label free quantification module integrated in MaxQuant.
	VIPER [84]	Visual Inspection of Peak/Elution Relationships.
Integrated platform	OpenMS [85]	A cross-platform software for the flexible analysis of MS data.
	Peaks Studio X + [86]	A peptide/protein identification & quantification software platform that offers complete solutions, including PTM and sequence variants.
	Skyline [87]	An open-source Windows client application for accelerating targeted proteomics experimentation.

**Table 3:** Downstream bioinformatics analysis software tools.

Category	Name	Description
Downstream bioinformatics analysis tools	COVAIN [182]	A software for statistics, time series, and correlation network analysis.
	CRONOS [137]	A cross-reference navigation server.
	GNET2 [179]	An R package for module inference of biological network.
	GSimp [123]	A Gibbs sampler-based left-censored missing value imputation approach for metabolomics studies.
	HIPPIE v2.0 [181]	A tool for enhancing meaningfulness and reliability of protein–protein interaction networks.
	IKAP [175]	A heuristic framework for inference of kinase activities from phosphoproteomics data.
	INGA [143]	A tool for protein function prediction combining interaction networks, domain assignments, and sequence similarity.
	KSEA [176]	A web-based tool for kinase activity inference from quantitative phosphoproteomics.
	Neglog [180]	Reconstruction of Human Protein–Protein Interaction Networks.
	Pathview [147]	An R package for pathway-based data integration and visualization.
	PP2A [163]	An integrated workflow for charting the human interaction proteome.
	ProLoc-GO [141]	Utilizing informative Gene Ontology terms for sequence-based prediction of protein subcellular localization.
	PSEA-Quant [149]	A Protein Set Enrichment Analysis on Label-Free and Label-Based Protein Quantification Data.
	viper [189]	Virtual Inference of Protein-activity by Enriched Regulon analysis.
Databases and web services for downstream analysis	STRING [190]	A database for quality-controlled protein-protein association networks.
	SIGNOR [135]	A database of causal relationships between biological entities.
	KEGG [148]	A disease and pathway database.
	PANTHER Pathway [145]	An Ontology-Based Pathway Database Coupled with Data Analysis Tools.
	Pathway Commons [185]	A web resource for biological pathway data.
	PhosphoSitePlus [134]	A comprehensive web services for post-translational modifications.
	PICR [136]	Reconciling protein identifiers across multiple source databases.
	Reactome [146]	A database of reactions, pathways, and biological processes.

## Abbreviations

MS	Mass spectrometry
FDR	False discovery rate
AP-MS	Affinity purification-mass spectrometry
DIA	Data-independent acquisition
PTM	Posttranslational modifications
LC-MS/MS	Liquid-chromatography coupled with tandem mass spectrometry
MALDI IMS	Matrix-assisted laser desorption/ionization imaging mass spectrometry
PSEA	Protein set enrichment analysis
GSEA	Gene set enrichment analysis
VSN	Variance stabilization normalization
HMM	Hidden Markov model
SVM	Support vector machines
LIMMA	Linear Models for Microarray Data
PSM	peptide spectrum match
ICAT	Isotope coded affinity tag
ICPL	isotope coded protein labeling
SILAC	stable isotope labeling by amino acids in cell culture
PPI	Protein-protein interaction
